# Future Development Strategy of Piezoelectric Devices

**DOI:** 10.3390/mi17010145

**Published:** 2026-01-22

**Authors:** Kenji Uchino

**Affiliations:** Electrical Engineering, The Pennsylvania State University, University Park, PA 16802, USA; kenjiuchino@psu.edu

Firstly, the five key development trends in the field of piezoelectric materials are discussed to offer the present perspective: “Performance to Reliability,” “Hard to Soft,” “Macro to Nano,” “Homo to Hetero,” and “Single to Multi-functional.” In the current materials trend, the accelerating development of Pb-free piezoelectrics for replacing conventional PZT is largely driven by the growing global regulatory focus on toxicity reduction [1,2,3]. Additionally, high-power piezoelectrics with low loss have emerged as a pivotal research area from the perspective of enhancing energy efficiency, signifying a paradigm shift from conventional approaches. This paradigm shift is characterized by a focus on “real (strain magnitude) to imaginary performance (heat generation reduction),” underscoring the significance of this transformation in the field [4,5]. Secondly, we are witnessing the resurgence of polymer technology in the post-1980s era, owing to the notable elastically soft superiority that these materials have demonstrated. The contemporary imperative for portable electronic devices is to incorporate human interfaces that are larger, thinner, lighter, and mechanically flexible. This demand has precipitated the development of elastically soft displays, electronic circuits, and speakers/microphones. Polymeric and polymer–ceramic composite piezoelectrics are undergoing a resurgence and commercialization [6]. Elastomers represent a class of alternative materials that have garnered significant interest for their potential applications in energy harvesting [7]. The focus on PZN-PT (or PMN-PT) single crystals stems from the discovery of their rubber-like-soft piezoelectric properties, which has persisted for over two decades [8,9,10,11]. In the domain of MEMS/NEMS, piezo MEMS represents a significant miniaturization target. This objective entails the integration of piezo-actuators within micro-scale devices, with the primary aim of facilitating bio/medical applications that contribute to the maintenance of human health [12,13]. Fourthly, the “homo to hetero” structural change is a recent research trend in the field. Stress-gradient in terms of space in a dielectric material exhibits piezoelectric-equivalent sensing capability (i.e., “flexoelectricity”) [14,15], while electric-field gradient in terms of space in a semiconductive piezoelectric can exhibit bimorph-equivalent flextensional deformation (“monomorph”) [16,17,18]. Finally, the realization of novel functions can be achieved through the integration of two effects. The development of magnetoelectric devices, in which a voltage is generated by applying a magnetic field, has been achieved through the laminating of magnetostrictive Terfenol-D and piezoelectric PZT materials [19,20]. The photostriction phenomenon has been demonstrated through the coupling of photovoltaic and piezoelectric effects in PLZT [21,22].

Next, we will proceed to examine the prospective development strategy. In the aftermath of World War II, the Japanese government adopted a four-Chinese-character strategy “重厚長大” in the 1960s, known as “heavier, thicker, longer, and larger.” This strategy entailed the development of heavier ships, thicker steel plates, longer buildings, and larger power plants (dams), with the objective of reconstruction and recovery from the devastation of WW II. Subsequently, a slogan diametrically opposed to the previous one emerged in the 1980s: “軽薄短小 (lighter, thinner, shorter, and smaller).” The evolution of technology has led to significant advancements in the field, with printers and cameras becoming more lightweight and compact. Concurrently, thinner computers and flat-panel televisions have gained popularity due to their portability and ease of use. The printing time and information transfer period have also been reduced, enhancing the efficiency of these devices. Furthermore, air conditioners and tape recorders, such as the “Walkman” by SONY, have undergone a reduction in size, contributing to a more compact and portable lifestyle. At the dawn of the 21st century, environmental degradation, resource depletion, and food scarcity had emerged as pressing concerns. The necessity of global regulations, also known as the Global Regime, has been increasingly emphasized. The government-initiated technology, often referred to as “politico-engineering,” has seen a resurgence in its importance, as it plays a crucial role in overcoming regulatory constraints. Concurrently, there has been an increase in both multinational terrorist attacks and global pandemic diseases.

Accordingly, the author posited a novel four-Chinese-character keyword for the contemporary epoch of “politico-engineering”: “協守減維” (cooperation, protection, reduction, and continuation). In the contemporary digital landscape, the imperative for global coordination and international cooperation in the standardization of internet systems and computer cables has become paramount. This necessity arises from the urgent need to accelerate the development and implementation of effective mutual communication systems: the Kyoto and Paris Protocols represent international agreements that are linked to the United Nations Framework Convention on Climate Change to reduce greenhouse gas emissions. It is imperative to ensure the protection of the territory and environment from the incursion of enemies or natural disasters, as well as the containment of the spread of infectious diseases. The reduction of toxic materials, such as lead, heavy metals, dioxin, and the reduction of resource and energy consumption, is also a key objective. Politico-Engineering encompasses two distinct categories of technologies: (1) legally regulated normal technologies, including sustainability, and (2) crisis technologies.

In addition to the aforementioned general demands, the author introduces leading actuator/sensor and piezoelectric technologies in this editorial, relating them to the “sustainability” and “crisis” technologies. The objective is to encourage further research expansion in this area. The establishment of a sustainable society necessitates the implementation of several key elements, including the utilization of non-toxic materials, such as lead-free piezoelectrics, the development of technologies for the disposal of existing hazardous materials, such as high-power ultrasonic transducers [2], the reduction of contamination gas, for instance through diesel injection valves [23], the creation of new energy sources, such as piezoelectric renewable energy harvesting systems [24,25,26], and the development of energy-efficient devices. Crisis technologies can be categorized into five distinct types: (a) natural disasters (e.g., earthquakes, tsunamis, tornadoes, hurricanes, and lightning); (b) epidemic/infectious diseases (e.g., smallpox, polio, measles, and HIV); (c) enormous accidents (e.g., the Three-Mile-Island core meltdown, the BP oil spill); (d) intentional accidents (e.g., acts of terrorism, criminal activity); and (e) civil war, war, and territorial aggression. The following devices, which are currently undergoing development, exemplify the corresponding aforementioned applications: (a) Magnetoelectric sensors were developed with a piezoelectric film sandwiched by magnetostrictive films. A minor fluctuation in the local earth magnetic field can serve as an indicator of imminent seismic activity [20]. (b) The Penn State group developed a portable hypochlorous-acid disinfection device with a piezoelectric ultrasonic humidifier to neutralize biological attacks, such as anthrax [27]. (c) The incident at the Fukushima Nuclear Power Plant in 2011, which resulted in a critical meltdown of the fuel rod, can be attributed to the absence of a comprehensive monitoring system. The Penn State group developed a high-temperature (600 °C) piezoelectric transducer with an AlN single crystal for monitoring the uranium rod condition in a nuclear chamber [28]. (d) and (e) In the 21st century, the concept of “green” weapons, which are environmentally friendly, has gained widespread acceptance. In this regard, “programmable air-burst munition” (PABM) was developed in 2004. The 25 mm caliber “Programmable Ammunition” was developed by ATK Integrated Weapon Systems and Micromechatronics [29].
